# Differential diagnosis of anterior sacral meningocele during the evaluation of post-hysterectomy pelvic collections

**DOI:** 10.1590/0100-3984.2015.0099

**Published:** 2016

**Authors:** Ronaldo Garcia Rondina, Richard Volpato, Luiz Felipe Alves Guerra, Diego Lima Nava Martins, Laís Bastos Pessanha

**Affiliations:** 1 Universidade Federal do Espírito Santo (UFES), Vitória, ES, Brazil.

**Figure 1:**
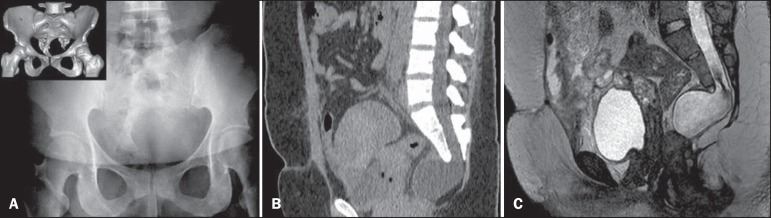
**A:** Conventional radiography showing unilateral sacral curvature (scimitar sacrum). Detail: Three-dimensional CT reconstruction for better characterization of the findings. **B:** CT with reformatting in the sagittal plane, showing a cyst-like formation with hypodense liquid content on the presacral space, apparently communicating with the spinal canal, dislocating the rectum to the right. **C:** MRI with sagittal slices in a T2-weighted sequence, showing morphological and structural alteration with sacral dysraphism, in which the dural sac is insinuated toward the presacral space, with homogeneous cerebrospinal fluid contents. It is also possible to observe the tethered spinal cord and conus medullaris at the L3 level.

*Dear Editor*,

Here, we present the case of a 34-year-old woman who suffered postoperative pain and fever after subtotal abdominal hysterectomy. Conventional radiography of the pelvis showed unilateral sacral curvature (Figure 1A). Computerized tomography (CT) performed on the second postoperative day revealed a dense loculated collection, interspersed with small air bubbles, in the pelvic cavity and a cyst-like formation with hypodense liquid content in the presacral space, communicating with the spinal canal, dislocating the rectum to the right (Figure 1B). A diagnosis of anterior sacral meningocele (ASM) was made, and the surgical team was informed of its coexistence with the postoperative pelvic collections. A new procedure was carried out to drain the collections, care being taken to avoid the sacculation caused by the ASM, which was visible and palpable. Magnetic resonance imaging (MRI) was carried out in order to monitor the postoperative drainage and to characterize the malformation in greater detail (Figure 1C).

Various conditions related to anomalies in central nervous system development have been reported in Brazil^(1-3)^. ASM is a rare form of spinal dysraphism, in which the meningeal sac herniates into the presacral space^(4,5)^. It accounts for approximately 5% of all retrorectal masses and is more prevalent in women^(6)^.

ASM can occur in isolation or in association with other congenital abnormalities, such as urogenital malformations, anorectal malformations, lipoma, teratoma, epidermoid tumor, and dermoid cyst^(7,8)^. Due to its occult nature, it is generally diagnosed in the second or third decades of life. It can be asymptomatic or present with nonspecific symptoms, such as constipation, urological symptoms, and, in rare cases, neurological symptoms^(9)^. The diagnostic investigation can include conventional radiography, ultrasound, CT, and MRI.

With conventional radiography, it is sometimes possible to observe a "scimitar sacrum", characterized by an unilateral sacral curvature, which is considered pathognomonic for ASM^(10)^. Abdominal ultrasound can show a retrovesical cystic lesion, unspecific to the method^(11)^. CT is an important tool, because it provides detailed information on associated bone alterations and can reveal herniation of the meningeal sac. MRI is the test of choice for evaluating ASM, because it creates high contrast between soft tissues, which makes it able to detect any communication between the ASM and the subarachnoid space, and provides detailed information about other related abnormalities that might be present^(4)^. However, when a communication with the subarachnoid space is narrow, MRI can fail to show it. In such cases, myelography with intrathecal injection of contrast can be necessary^(12)^.

The differential diagnosis of ASM includes cystic lesions located in the presacral region^(7,13)^: tumors of the gastrointestinal or genitourinary tract; epidermoid or dermoid cysts; aneurysmal bone cyst; hamartoma; hydatid cyst; lipoma; lymphangioma; perineural cyst; rectal duplication cyst; gynecologic tumors; teratoma; or teratocarcinoma. The most important means of establishing the definitive diagnosis is detecting communication between the cystic lesion and the subarachnoid space^(11)^.

In the case in question, making the diagnosis of ASM was particularly important because the patient underwent laparotomy to drain the hemorrhagic collections in the pelvis. During that procedure, an unwarranted, inadvertent intervention in the meningocele could have had disastrous consequences.
